# Referring Adolescent Primary Care Patients to Single-Session Interventions for Anxiety and Depression: Protocol for a Feasibility Study

**DOI:** 10.2196/45666

**Published:** 2023-08-09

**Authors:** Mara Eyllon, Michelle Dalal, Laura Jans, Ian Sotomayor, Gabrielle Peloquin, James Yon, Rochelle Fritz, Jessica Schleider

**Affiliations:** 1 Practice Research Network Reliant Medical Group Worcester, MA United States; 2 Northeastern University Northeastern University Health and Counseling Services Boston, MA United States; 3 Department of Pediatrics Chan School of Medicine University of Massachusetts Worcester, MA United States; 4 Department of Psychology Stony Brook University Stony Brook, NY United States; 5 Behavioral Health Department Reliant Medical Group Worcester, MA United States

**Keywords:** adolescents, behavioral health care, mental health, primary care, single-session interventions

## Abstract

**Background:**

Despite the growing prevalence of depression and anxiety among adolescents, fewer than half access appropriate mental health care. Single-session interventions (SSIs) for depression and anxiety offered in primary care are a promising approach to bridging the treatment gap.

**Objective:**

We aimed to implement a clinical workflow for primary care and behavioral health providers to refer patients aged 13 to 17 years with mild to moderate depression and anxiety symptoms to Project YES (Youth Empowerment and Support), an open-access SSI platform, in a large group medical practice with an integrated behavioral health department.

**Methods:**

Pediatric primary care and integrated behavioral health providers will be educated on the benefits of Project YES for adolescent anxiety and depression and trained in a workflow integrated within the electronic health record system, Epic, to refer patients during well-child visits and pediatric behavioral health visits. Patients with mild to moderate internalizing symptoms based on the 17-item Pediatric Symptom Checklist or youth Pediatric Symptom Checklist will be invited to try an SSI through Project YES. We will examine provider uptake and perceptions of acceptability, feasibility, and appropriateness over time.

**Results:**

The rollout will take place between November 2022 and May 2023, when outcomes will be evaluated. Data analysis and manuscript writing are anticipated to be completed during the summer of 2023.

**Conclusions:**

SSIs such as those available through Project YES have the potential to provide low-cost, evidence-based mental health treatment to adolescents with mild to moderate depression and anxiety. If deemed feasible and acceptable, providing SSIs in primary care settings could significantly improve access to mental health care without taxing pediatric primary care and behavioral health providers.

**International Registered Report Identifier (IRRID):**

DERR1-10.2196/45666

## Introduction

### Overview

Among adolescents, the incidence of depression and anxiety has increased significantly over the past decade. In 2019, 37% of adolescents (in the United States) experienced symptoms of sadness or hopelessness, while 21% received a diagnosis of major depressive disorder and 9% were diagnosed with an anxiety disorder [1]. Exacerbating the crisis in youth mental health, the COVID-19 pandemic led to unparalleled disruption in the day-to-day lives of youth and adolescents. School and social events were put on hold, and they observed the consequences of COVID-19 in their families and communities, resulting in a 2-fold increase in depression and anxiety [2].

Despite the rising prevalence of mental health concerns among adolescents, 75% of adolescents with mental health conditions do not receive appropriate care [2] due to a lack of access and acute shortages of qualified pediatric mental health clinicians [2]. Primary care providers are increasingly called upon to identify and intervene with mental health concerns [3], and it is therefore critical for pediatric primary care providers to have effective tools to offer to patients and their families to help manage symptoms. However, such resources are often not available to primary care providers, and patients often wait months before receiving care from licensed mental health professionals following a referral from primary care. Self-guided and web-based single-session interventions (SSIs) are an effective treatment for adolescents with mild to moderate mental health concerns [4] and may provide a stopgap for patients in need of more intensive care who are waiting to receive services. This paper presents the protocol for a novel clinical process for pediatric and integrated behavioral health care providers to refer adolescent patients to use the SSI platform Project YES (Youth Empowerment and Support) at a large group medical practice with an integrated behavioral health department.

### Access to Mental Health Care Among Adolescents

In 2021, the surgeon general declared a mental health emergency, prompting responses from multiple sectors to allocate resources to pediatric mental health care [5]. Yet, despite such calls, the population-level mental health of adolescents has worsened throughout the pandemic as growing patient needs are compounded by acute shortages of qualified providers [2,6]. While more than half of all adolescents with mental health concerns do not receive any treatment, even among those who are treated, most do not complete a full course of evidence-based treatment and rarely attend more than 3 to 4 sessions [7]. Beyond the issue of supply and demand, barriers to accessing and completing a clinically appropriate course of treatment for mental health concerns are many. Among adolescents, stigma, shame, and beliefs about who seeks mental health care may inhibit adolescents from accessing necessary interventions [8]. Confidentiality from parents is another important barrier and the Adolescent Health Care Barrier Survey revealed that nearly 40% of adolescents reported forgoing mental health services because they did not want their parents to know [9]. These barriers highlight the need for evidence-based tools which are both acceptable and directly accessible to youth presenting with mood- and anxiety-based symptoms.

SSIs are designed to be intentionally brief, flexible, and potent. Digital SSIs, which can be web-based and self-guided, are a sustainable and low-cost means of disseminating evidence-based treatments at scale [4,10]. SSIs do not rely on patient-provider ratios and can be offered to many patients by just a few providers in under 5 minutes. Their broad accessibility can reduce stigma around mental health concerns and help to normalize the use of appropriate care, whether a patient is going through a challenging time or working through a persistent mental health condition. Furthermore, SSIs can be disseminated in a variety of settings, such as schools and through social media [10,11], and adolescents can use SSIs with confidentiality from their parents. Despite the demonstrated efficacy of SSIs disseminated through social media, they are seldom made available in pediatric health care settings. As digital technologies are increasingly leveraged to close the gap in mental health care, SSIs may provide an additional resource for providers to deploy to adolescent patients.

### Evidence for SSIs

Given their demonstrated effectiveness and flexible nature, web-based SSIs allow youth to access mental health support when and where they need it. A growing body of evidence suggests that SSIs hold the potential to reduce a variety of mental health problems. In a meta-analysis of 50 randomized controlled trials targeting youth mental health issues, SSIs were effective (Hedges *g*=0.32) in improving several clinically relevant outcomes [12]. Notably, SSI effectiveness did not significantly differ between therapist-administered and self-administered interventions. In addition, shorter (<60-minute) interventions were found to be as effective as longer SSIs [12].

In a randomized controlled trial, a virtual, self-administered growth mindset intervention reduced depression symptoms in high-symptom youth 9 months after completion [13]. Other trials of digital SSIs teaching growth mindsets similarly demonstrated their potential to reduce depression symptoms [14-16]. In a diverse group of adolescents recruited from all 50 US states, 2 SSIs improved youth agency immediately postintervention and decreased anxiety, depression, hopelessness, and restrictive eating behaviors 3 months later [17]. Importantly, these SSIs appear to be similarly effective for youth of diverse racial-ethnic identities, gender identities, subjective family social status, and sexual orientations [18].

Web-based SSIs have been evaluated in a more naturalistic setting through an open-access web-based platform called Project YES [19]. Project YES allows adolescents (aged 11 to 17 years) to choose from 3 web-based interventions, each of which spans approximately 30 minutes and includes a core element of evidence-based treatment [10]. Youth may choose from Project Personality (which teaches that personality traits can change), Project CARE (which centers on the benefits of self-compassion), or the Action Brings Change (ABC) project (which encourages youth to engage in activities to improve their mood). Youth can complete as many interventions as they would like, as often as they choose to, and entirely free of charge. In a pilot trial, adolescents who completed an intervention through the Project YES platform experienced immediate improvements in hopelessness, self-hatred, perceived control, and agency. In addition, youth rated the interventions as acceptable, and 34.32% of youth who started an intervention completed it [10]. This surpassed completion rates for other digital interventions in naturalistic settings (1%-28%) [20].

Project YES has also been culturally adapted and translated into Spanish for youth living in San Antonio, Texas. Through a series of semistructured focus groups, San Antonio youth stakeholders shared their lived mental health experiences and co-designed the adapted interventions [21]. San Antonio youth who completed an intervention in English experienced immediate improvements in hopelessness, self-hatred, and perceived agency. Similarly, youth who completed a culturally adapted and translated intervention experienced immediate improvements in self-hatred [21]. The completion rate was 49.6%, and the interventions were rated as acceptable by youth [21].

Each SSI in Project YES includes shared core elements thought to underlie its effectiveness. Namely, the interventions (1) present youth with brain science to validate the concepts introduced in the activity, (2) empower youth by placing them in an “expert” role, (3) promote retention of the material with “saying is believing” exercises, and (4) include vignettes from peers and valued others [19]. Conceptually, the SSIs operate by shifting proximal beliefs or behaviors (eg, hopelessness, agency, expectancy that change is possible), which are thought to spark behavior change underlying a longer-term reduction of internalized symptoms [17,22]. SSIs are designed with a few key assumptions: (1) something good can come in 1 session, and 1 session may be the last; (2) change can, and is expected to, occur in the moment; (3) and individuals already have the inner capacity to alter their thoughts, emotions, and behaviors to create meaningful change [23]. Altogether, web-based SSIs, such as those in Project YES, are promising options for delivering brief, evidence-based mental health support with the potential to fill need-to-access gaps [24] for youth experiencing mental health problems.

Although SSIs disseminated through social media have been explored in other contexts, less is understood about how to optimize their implementation in primary care settings and integrate them into existing clinical workflows. Identifying best practices for integrating SSIs into current pediatric provider workflows may give providers an additional resource to recommend to their patients experiencing symptoms of anxiety or depression. To this end, the primary objective of this quality improvement pilot is to establish the feasibility and acceptability of delivering a SSI to adolescents with mild to moderate depression and anxiety in pediatric primary care and behavioral health.

## Methods

### Setting

Reliant Medical Group (RMG) is a multispecialty group medical practice in Massachusetts. With 19 clinics, RMG provides services to 240,000 patients per year, including 64,000 pediatric patients, 29% of whom have been diagnosed with a mental health condition. RMG has an integrated behavioral health department in which patients are triaged by a licensed mental health care provider from primary care to integrated behavioral health services within RMG or in the community. The pediatric behavioral health team has 13 providers, including psychologists, master’s level clinicians, nurse practitioners, and psychiatrists who handle prescribing needs. However, the integrated model only serves one-third of the patient population. In 2021, a total of 1910 pediatric patients who were not appropriate matches for the integrated care model were referred outside of RMG to receive services in the greater community. RMG also has a practice research network that includes a team of research scientists and coordination staff to oversee research studies and evaluate quality improvement initiatives. This protocol was developed by a multidisciplinary team including a research scientist, pediatrician, pediatric psychologist, and pediatric psychiatrist in collaboration with the Lab for Scalable Mental Health at Stony Brook University.

### Study Design

This paper describes a quality improvement pilot designed to evaluate the feasibility and acceptability of disseminating Project YES in primary and behavioral health care, as well as evaluate short-term changes in mental health outcomes among participants.

### Patient Screening

**Figure 1:**
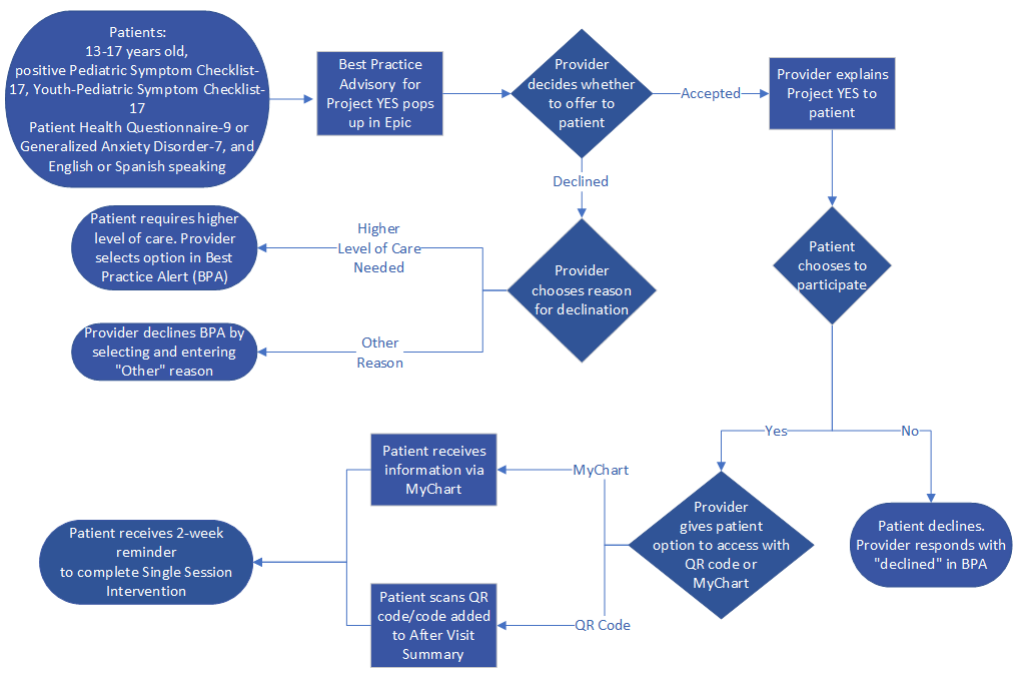
Clinical workflow for referring patients to Project YES.

Figure 1 displays the clinical workflow for screening and referring patients to Project YES. First, as part of routine mental health screening during well-child visits, pediatric primary care and behavioral health providers or their medical assistants administer either the 17-item Pediatric Symptom Checklist (PSC-17) [25] or the Youth PSC-17 (Y-PSC-17) [25] to all patients aged between 13 and 17 years. When the total internalizing symptom score is elevated on either of these scales, follow-up screeners such as the Patient Health Questionnaire-9 (PHQ-9) [26] and the General Anxiety Disorder-7 (GAD-7) [27] are administered. Information from these screeners is used to determine whether patients meet criteria for a clinical diagnosis on the spectrum of depression or anxiety and whether routine follow-up is necessary. The PHQ-9 and GAD-7 screeners are also routinely administered as independent screening tools by behavioral health providers as part of the behavioral health visit. Providers will have the opportunity to refer patients to Project YES when the following criteria in Textbox 1 are met:

**Inclusion and exclusion criteria.**

**Inclusion criteria**

- Patient is aged between 13 and 17 years.
- Patient meets at least one of the following criteria at the visit or in the preceding 3 months: (1) Patient Health Questionnaire-9 (PHQ-9) score >5; (2) Y-PSC-17 or PSC-17 internalizing score ≥5; (3) total Youth 17-item Pediatric Symptom Checklist (Y-PSC-17) or 17-item Pediatric Symptom Checklist (PSC-17) score ≥15; and (4) General Anxiety Disorder-7 (GAD-7) score ≥5.
- Comfortable speaking and reading in either English or Spanish.

**Exclusion criteria**

- Active suicidal ideation (score ≥1 on question 9 of the PHQ-9, if completed).

### Best Practice Alert

**Figure 2:**
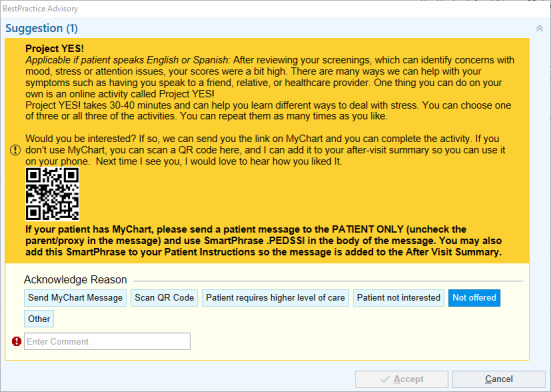
Best practice alert screen.

When a patient meets the eligibility criteria, a best practice alert (BPA), displayed in Figure 2, will be triggered in Epic, RMG’s electronic health records (EHR) system. The BPA pop-up screen provides instructions for referring a patient to Project YES, including a brief overview of Project YES, its potential benefits, and how to access it. If the patient indicates that they are interested, the provider can offer SSI in 1 of 2 ways:

1. Send the patient a QR code or a link to participate in the SSI through MyChart (the patient front-end of Epic). The MyChart message will include a link to Project YES at the Lab for Scalable Mental Health website and instructions for participating.
2. Provide the patient with a QR code displayed in the BPA that patients may scan using their mobile device, allowing for the retrieval of Project YES for later use. The QR code is also included in the after-visit summary for later use. The QR code will direct patients to Project YES at the Lab for Scalable Mental Health website.

### Educating Providers on Project YES

First, to ensure representation from participating departments, the implementation team included a pediatrician from the pediatrics department and a pediatric clinical psychologist and pediatric psychiatrist from the behavioral health department. Initial permission and buy-in were sought from the key leadership, including the chief of pediatrics and chief of behavioral health, before designing the workflow. The SSI workflow is targeted toward pediatric and behavioral health providers, including pediatricians, nurse practitioners, clinical psychologists, and master’s-level mental health clinicians. All pediatric medical providers and integrated behavioral health providers will be given a brief presentation by members of the implementation team at monthly department meetings, 4 weeks before launching the workflow, and again the week before launch. The introductory presentation covers the gap in care that SSIs can fill, the core elements of Project YES and its 3 interventions, an example of 1 of the SSIs, the evidence base for Project YES, and instructions for referring patients to Project YES during care. Monthly check-ins at department meetings will be held to review processes, answer questions, and gather assessments as well as informal feedback. The monthly check-ins are led by the pediatric behavioral health lead, who is available to answer any questions and address and track comments or concerns. Her contact information is also provided on all materials in the event a provider has questions in real-time.

Each unique clinic also holds individual site meetings where the behavioral health lead will reiterate the core components of Project YES to the staff and providers at each individual clinic. This will also provide the lead an opportunity to introduce new clinical team members to the intervention and referral processes during their orientation. To allow for broader dissemination of information, a monthly behavioral health newsletter sent to all staff in behavioral health and pediatrics will also contain information about the intervention. Lastly, additional materials, such as the PowerPoint slides and other informational materials, will be posted to department shared drives for on-demand review.

### Patient Participation

Patients will receive an invitation to participate with a link to the SSI and instructions for participating through MyChart (Multimedia Appendix 1). The patient will click on the link in their MyChart messages that will take them to the Lab for Scalable Mental Health’s website. They will be asked to select the module they want to participate in and indicate that they are aged between 13 and 17 years and a patient at RMG in Massachusetts before beginning. The patient will not enter any potentially identifying information. By entering that they are an RMG patient, the Lab for Scalable Mental Health will be able to filter RMG’s data for the program evaluation. Before and after completing the module, the patient will be invited to complete a series of anonymous, optional questionnaires within Project YES’ Qualtrics interface. In the event a participant in Project YES experiences distress, the Project YES platform provides a “Need help now?” button on the main website and on each page of the platform that leads to [28]. The linked page provides immediately accessible crisis resources such as the Crisis Text Line, the National Suicide Prevention Lifeline, and a Hotline for LGBTQ+ Youth.

### Data Collection

Provider referral records will be collected through RMG’s EHR, and anonymous patient engagement and outcomes data will be collected within Project YES’ Qualtrics interface by the Lab for Scalable Mental Health and sent to RMG for analysis. Table 1 describes the specific data to be collected and the corresponding methods of analysis.

**Table 1:** Overview of data collected and analytic methods.

Data	Items	Analytic methods	
Behavioral health and primary care providers: EHR^a^ data	Number of eligible patients seen. Number of referrals made. Reasons indicated for not providing SSI^b^ (patient inappropriate, not interested, or other). Number of patients selecting QR code versus MyChart message access.	Descriptive statistics: proportion of providers in each department offering SSI.	
Patients: engagement metrics and feedback	Number of RMG^c^ patients who entered the site. Total number of patients who completed the SSI. Program Feedback Scale		Descriptive statistics.
Providers: data collected at monthly behavioral health and pediatric department meetings	Acceptability of Implementation Measure (AIM). Feasibility of Implementation Measure (FIM). Intervention Appropriateness Measure (IAM).	Descriptive statistics. Repeated measures ANOVA.	

^a^EHR: electronic health record.

^b^SSI: single-session intervention.

^c^RMG: Reliant Medical Group.

### Provider Measures

A total of 3 implementation outcome measures will be collected from providers to evaluate the implementation of Project YES. Each outcome measure consists of 4 items rated on a 5-point Likert scale ranging from “completely disagree” to “completely agree.” The scales demonstrate high reliability, with Cronbach α reported between .85 and .91, structural validity indicated by factor loadings between 0.75 and 0.89, and test-retest reliability coefficients between 0.73 and 0.88 [29].

#### Acceptability of Intervention Measure

The Acceptability of Intervention Measure (AIM) measures the appeal of an intervention to target providers (eg, Project YES meets my approval) [29].

#### Intervention Appropriateness Measure

The Intervention Appropriateness Measure (IAM) measures whether an intervention is fitting and relevant to the target participants within a given setting (eg, Project YES seems applicable) [29].

#### Feasibility of Implementation Measure

The Feasibility of Intervention Measure (FIM) measures whether implementing a given intervention is achievable within the setting given the time and resources required to carry it out successfully (eg, Project YES seems doable) [29].

### Participant Measures

The following measures are the same as those described in previous reports on Project YES; however, in the context of this data collection, we include both the English and Spanish versions. Additionally, this study implemented a single-item question in both English and Spanish surveys, asking whether the participant was a patient with RMG. Only data from participants who select yes to confirm patient status with RMG and report being between the ages of 13 and 17 years will be included in the final analysis.

#### Demographics

Participants will identify their age range (provided in ranges to maintain anonymity: 10 years or younger, 11-12 years, 13-15 years, 16-17 years, 18 years or older), sex assigned at birth, gender identity, sexual orientation, race or ethnicity, how they learned about Project YES, and whether they are patients at RMG in Massachusetts.

#### Program Feedback Scale

The Program Feedback Scale (PFS) [30], which is routinely used to evaluate acceptability and user perceptions of SSIs [4,21,23,31] asks participants to rate agreement with 7 statements indicating perceived acceptability and feasibility of their selected SSI (eg, “I enjoyed the program”) on a 5-point Likert scale (1=“really disagree;” 5=“totally agree”). The scale was adapted from existing, validated acceptability assessments of digital interventions; adaptations from existing scales were necessary to exclude items that are inapplicable to web-based SSIs (eg, items referencing frequency of use or interest in continuing to revisit the program). The PFS will also assess participants’ open-response feedback. PFS item scores may be evaluated individually or through a mean score across items. Mean responses to each PFS item will be evaluated separately to gain insight on the acceptability in specific domains (eg, ease of use and understanding, enjoyability).

### Ethical Considerations

This quality improvement project was exempted from review by the Optum Office of Human Subjects Research Institutional Review Board (IORG0010356) as it was deemed not human subjects research.

## Results

The program was initiated in November 2022 at 1 site to ensure Epic components functioned as intended and then expanded across all sites in December 2022. The program will continue for 6 months before the program evaluation will commence; however, referral, implementation assessment, and patient engagement data will be reviewed iteratively to identify any adaptations that need to be made. Data analysis and manuscript writing are expected in the summer of 2023.

## Discussion

### Overview

Closing the gap in pediatric mental health care requires rethinking how care is delivered and innovative models to overcome the common barriers of face-to-face therapy while delivering effective, evidence-based treatments. Calls for innovation in mental health care have been made [32,33] and COVID-19 has catalyzed the development of digital technology for mental health [34,35]. Such technology is not often available in primary care settings. Single-session treatments, which provide a single session of face-to-face psychotherapy to patients, have been explored in mental health care settings, reporting medium to large effect sizes [36-38], although they are not as commonly deployed in primary care settings. Importantly, compared to single-session treatments, SSIs may be self-guided and rapidly disseminated among patients by providers without special training. Given that the modal number of psychotherapy sessions patients attend is 1 [7], while a minimum of 12-20 sessions may be required for symptom resolution, SSIs developed with the explicit intention of resolving symptoms in just 1 session are not only more accessible but may also be a more appropriate approach to improving treatment for adolescent mental health conditions. In addition to being a front-line intervention for primary care providers, SSIs may also be leveraged by behavioral health providers and offered as adjunctive or follow-up care for traditional face-to-face therapy.

### Strengths and Limitations

Offering patients SSIs in primary care and behavioral health settings is an efficient and cost-effective approach to narrowing the treatment-to-service gap in adolescent mental health care. Approaching this as an implementation project will allow us to understand the feasibility, acceptability, and appropriateness of SSI delivery in a pediatric primary care and integrated behavioral health setting and thus identify potential barriers and facilitators providers may face when offering Project YES to their patients. This information may be used to iteratively adapt the clinical workflow described to streamline the process for providers and patients alike, thus improving uptake. This protocol was developed in close collaboration with a pediatric clinical team and feedback from leadership to ensure that it is relevant, efficient, and can be easily grasped and deployed by providers. Further, making the SSIs accessible to patients with a wide range of symptoms may help reduce stigma around mental health care. Finally, adolescents who may otherwise forgo behavioral health services due to concerns about their confidentiality from their parents can use the SSIs privately without their parents being notified.

Nonetheless, there are several limitations to the study design. Participation is contingent on elevated scores on the screener questionnaires. However, patients who may not meet clinical criteria may also benefit from the SSIs, which can be effective for subclinical internalizing symptoms. To address this, future iterations of this project may explore expanding referrals to all adolescent patients, regardless of meeting screening criteria, or advertising the program on waiting room monitors and in other patient-facing materials. Additionally, this work is only being piloted at a single-group medical practice in Massachusetts and may not be generalizable to other populations. Furthermore, RMG has a predominantly White, English-speaking patient population. Although the intervention is available to Spanish-speaking patients, more work may be needed to understand its feasibility and acceptability in other clinical contexts and among diverse patient populations. Finally, without the use of a placebo-control group to control for potential effects of participating in an SSI in and of itself, the effectiveness data from this study may overestimate potential improvements in symptoms.

### Conclusion

While this work explores the dissemination of SSIs in primary care and pediatric behavioral health, SSIs have great potential as a proactive approach to the prevention of mental health concerns. Future work could explore the provision of SSIs to a wider range of adolescents in primary care to examine their potential upstream preventive effects. Furthermore, previous research indicates that parents of adolescents may also benefit from SSIs that are as brief as 15 minutes [4]. While the present workflow was designed to be accessed confidentially by adolescents, SSIs may be effectively disseminated to adolescent parents in primary care settings to raise their expectations for treatment, thus improving patient engagement [4].

SSIs are highly accessible and potent interventions that effectively help to fill the adolescent mental health treatment gap at low costs and require minimal provider time and resources. We anticipate that referring patients to Project YES will be both feasible and acceptable for primary care providers, and its use will be acceptable and effective for patients.

## Appendix

Multimedia Appendix 1 MyChart message to patients who elect to participate.

## Abbreviations

ABC: Action Brings Change
AIM: Acceptability of Intervention Measure
BPA: best practice alert
EHR: electronic health record
FIM: Feasibility of Intervention Measure
GAD-7: General Anxiety Disorder-7
IAM: Intervention Appropriateness Measure
PFS: Program Feedback Scale
PHQ-9: Patient Health Questionnaire-9
PSC-17: 17-item Pediatric Symptom Checklist
RMG: Reliant Medical Group
SSI: single-session intervention
YES: Youth Empowerment and Support
Y-PSC-17: Youth 17-item Pediatric Symptom Checklist
