# Supplementary material for: Referring Adolescent Primary Care Patients to Single-Session Interventions for Anxiety and Depression: Protocol for a Feasibility Study
Source: JMIR Res Protoc. 2023 Aug 9;12:e45666. doi: 10.2196/45666 (PMC10448284; doi:10.2196/45666)
Supplement: Multimedia Appendix 1 [file resprot_v12i1e45666_app1.docx]

Appendix 1. MyChart message to patients who elect to participate.

| Thank you for your interest in participating in Project YES Your primary care or behavioral health provider believes you may benefit from Project YES!  Project YES! is a 30 to 40-minute self-guided online program which takes you through different activities to help with any feelings of stress, depression, or anxiety that you may be experiencing. You may choose to participate in any of the three programs Project CARE, Project Personality, or the ABC Project. You may try as many different programs as you like and you may repeat them as many times as you want.  Please click Project YES To go to the modules. Link goes to --> https://stonybrookuniversity.co1.qualtrics.com/jfe/form/SV_08OCfczeMJ9l7mZ **Be sure to select that you are a patient at Reliant Medical Group in Worcester, Massachusetts.** This will help us to understand how the program benefits our patients. No personally identifiable information about you will be collected. z  If you have any questions, please reach out to your primary care or behavioral health provider. If you are experiencing an emergency please contact 911. |
| --- |
